# Investigating the Presence and Genetic Variability of Porcine Circovirus Types 2 and 3 in Live Markets in Border Cities of Northeast China

**DOI:** 10.1155/tbed/5526645

**Published:** 2025-06-19

**Authors:** Fanqi Sun, Meng Li, Shubo Li, Zhen Yang

**Affiliations:** ^1^Key Laboratory of Animal Diseases Diagnostic and Immunology, Ministry of Agriculture, MOE International Joint Collaborative Research Laboratory for Animal Health and Food Safety, The Belt and Road International Sci-Tech Innovation Institute of Transboundary Animal Disease Diagnosis and Immunization, College of Veterinary Medicine, Nanjing Agricultural University, Nanjing 210095, China; ^2^Liaoning Center for Animal Disease Control and Prevention, Shenyang 110164, China

**Keywords:** border, detection, live markets, porcine circovirus

## Abstract

Bordering cities and farms serve as potential hotspots for the transboundary spread of animal infectious agents. This study aimed to investigate the presence and genetic variability of porcine circovirus types 2 (PCV2) and 3 (PCV3) in live markets across six border cities (Dandong, Ji'an, Hunchun, Mishan, Fuyuan, and Heihe) in northeast China. Samples from pork (*n* = 44), cutting boards (*n* = 46), and meat stall floors (*n* = 42) were collected in 46 meat stalls. Quantitative PCR analysis detected PCV2 in 75.0% (95% CI: 59.7%−86.8%) of pork samples, 73.9% (95% CI: 58.9%−85.7%) of cutting board swabs, and 64.3% (95% CI: 48.0%−78.4%) of meat stall floor swabs. For PCV3, the detection rates in pork, cutting board swabs, and meat stall floor swabs were 31.8% (95% CI: 18.6%−47.6%), 67.4% (95% CI: 52.0%−80.5%), and 54.7% (95% CI: 38.7%−70.2%), respectively. Subsequent sequencing of positive samples identified five open reading frame (ORF)2 sequences of PCV2 from markets in Dandong, Fuyuan, and Hunchun, with one sequence from Dandong shared 99.4% homology with a Russia sequence. Similarly, five ORF2 sequences of PCV3 were obtained from samples in Hunchun, Heihe, and Ji'an, including a sequence from Hunchun showing 99.6% homology to a sequence from a pig farm in Changchun in Jilin Province. These findings suggest that border market pork trade may contribute to the introduction and dissemination of PCV2 and PCV3. The observed genetic similarities highlight potential transboundary transmission routes, emphasizing the need for active surveillance and control measures to mitigate the risks associated with the transboundary transmission of emerging swine pathogens.

## 1. Introduction

Porcine circovirus types 2 (PCV2) and 3 (PCV3) are nonenveloped and single-stranded circular DNA viruses of the genus *Circovirus* within the family Circoviridae [1]. PCV2 was first identified in Canada in 1991 and has since been reported worldwide [2–6]. It is a major pathogen in the swine industry, associated with substantial economic losses primarily due to its involvement in postweaning multisystemic wasting syndrome (PMWS) and other systemic diseases [7–9]. PCV2 exhibits rapid evolutionary dynamics characterized by elevated nucleotide substitution rates that drive substantial genomic diversification. The current classification system delineates eight major genotypes (PCV2a–PCV2h) through a principal criterion: maximum within genotype *p*-distance of 13%, minimum cluster internal node bootstrap support of 70%, and at least 15 identified sequences [10]. Recent studies highlight the emergence of PCV2d as the predominant genotype in China, replacing earlier strains like PCV2b [11–13]. A systematic review of PCV2 prevalence in China from 2016 to 2019 reported a pooled prevalence of PCV2 of 46%, with the highest regional prevalence in northeast China at 58.1% [14].

PCV3 was first identified in the United States in 2016 and has since been widely detected in countries such as China, South Korea, and Russia [15–19]. The virus has been linked to reproductive disorders and multisystemic inflammation [1, 17, 18, 20–23]. Currently, there is no consensus on the genotyping of PCV3 due to the use of independent classification criteria and schemes by different research groups. A consensus among a group of experts classified PCV3 strains into two genotypes—PCV3a and PCV3b—based on the following thresholds: a bootstrap support (or posterior probability) >0.9, a maximum genetic distance of 3% for the complete genome and 6% for the open reading frame (*ORF2)* gene, and concordant phylogenetic clustering between *ORF2* and the complete genome [24].

The genomes of PCV2 and PCV3 are approximately 1766–1768 nucleotides and 2000 nucleotides long, respectively. Both viruses share similar genomic structures, with ORFs (*ORF1* and *ORF2)* encoding the Rep and Cap proteins, which are involved in viral replication and modulation of the host immune response [1, 2, 17, 20, 21, 25, 26]. The *ORF2* gene demonstrates substantial genetic variability, particularly in PCV2, which is influenced by several contributing factors such as vaccination pressure, contact between wild and domestic pig populations, genotype competition, and global trade dynamics [27]. These genetic alterations may result in antigenic drift and notable antigenic differences within genotypes [28]. Previous studies have demonstrated that transboundary livestock trade facilitates the co-circulation of different viral genotypes such as African swine fever virus and avian influenza virus [29–31], thereby altering viral pathogenicity and amplifying the potential for adaptive evolution through reassortment events or recombinant strain emergence [32]. Given its high mutation rate among DNA viruses, PCV2 may serve as a model pathogen to assess whether frequent international trade activities in routinely monitored border cities contribute to viral genetic variation and the emergence of novel subtypes.

A paucity of epidemiological data exists regarding PCV2 and PCV3 dynamics in northeastern Chinese border cities, which serve as critical nodes for potential cross-border transmission. Certain border cities experience frequent cross-border movement due to the implementation of visa-free policies, increasing the risk of viral dissemination through human and trade activities. Notably, despite enhanced border inspections, trade restrictions, and administrative controls, persistent smuggling of live animals and animal-derived products from low-cost production zones to high-demand markets continues to amplify viral dissemination risks [33, 34]. Live markets in northeastern Chinese border cities—aggregation points for both locally sourced swine products and diverse human populations—represent ideal sampling sites for pathogen surveillance.

This study examines the prevalence and genetic variability of PCV2 and PCV3 in live markets across six northeastern Chinese border cities—Dandong, Ji'an, Hunchun, Mishan, Fuyuan, and Heihe—to assess contamination levels and cross-border transmission risks. In contrast to previous studies that focused on inland farms or slaughterhouses [11, 35, 36], this work emphasizes border-region markets, which serve as critical yet understudied nodes for pathogen dissemination. By analyzing detection rates, genetic diversity, and strain homology, the study provides novel insights into viral dynamics in high-risk zones and offers actionable evidence to enhance biosecurity strategies and curb the transnational spread of emerging swine pathogens.

## 2. Material and Method

### 2.1. Sample Collection

In July 2023, a total of 44 pork samples, 46 meat stall cutting board swabs, and 42 meat stall floor swabs were collected from live markets in six cities: Dandong, Ji'an, Hunchun, Mishan, Fuyuan, and Heihe. These cities span the three northeastern provinces of China (Liaoning, Jilin, and Heilongjiang Provinces), which border Democratic People's Republic of Korea and Russia. A two-stage sampling method was employed. First, all active live markets in each city were identified using DianPing.com, a widely used e-commerce and business directory in China. Next, one of the largest markets in each city was selected based on market size, daily visitor traffic, the number of meat stalls, and geographic accessibility to ensure representativeness as a major pork distribution hub. All selected markets strictly adhered to the live markets management policy in China [37]. At each market, meat stalls were selected for sampling. If there were fewer than 10 meat stalls, samples were collected from all of them. If there were more than 10 meat stalls, 10 stalls were selected for sampling using a convenient sampling method. From these selected stalls, pork meat, cutting board swabs, and meat stall floor swabs were collected. All meat stall owners consented to the sampling. To prevent contamination, each sample was packaged individually, and samples from each stall were kept separate. The collected samples were immediately placed in ice-packed coolers and shipped to the Veterinary Diagnostic Laboratory of Nanjing Agricultural University (NAU VDL) for PCV2 and PCV3 testing. The locations of the sampling cities were marked on a map using QGIS 3.28.0 (Open Source Geospatial Foundation Project, http://qgis.osgeo.org).

### 2.2. DNA Extraction

Each pork sample was minced using autoclaved scissors and diluted at a tenfold dilution with phosphate-buffered saline (PBS; 0.1 M, pH 7.4) mixed with steel beads. The mixture was placed in a rapid grinder apparatus (Jingxin, Shanghai, China) for grinding under the conditions of 60 Hz for 90 s for nine cycles. The ground pork samples were subjected to three freeze–thaw cycles, followed by centrifugation at 5000 × g for 15 min and the supernatant was collected. For cutting board swab and meat stall floor samples, the cotton swabs used for sampling were soaked in PBS, and the eluate was collected. DNA was extracted from the three processed samples using the DNA/RNA Extraction Kit (Vazyme, Nanjing, China), according to the manufacturer's instructions.

### 2.3. TaqMan qPCR

Based on previous studies, TaqMan qPCR was used to detect PCV2 and PCV3 [38, 39]. The reaction system for PCV2 included 10 μL of 2 × Premix Ex Taq Probe qPCR, 0.8 μL of forward and reverse primers (10 μM; Table 1), 0.4 μL of PCV2-probe (10 μM), 2 μL of the template, and ddH_2_O to a final volume of 20 μL. The amplification program was 95°C for 30 s, 95°C for 5 s, and 60°C for 30 s, repeated for 40 cycles.

For PCV3, the reaction system included 10 μL of 2 × Premix Ex Taq Probe qPCR, 1.2 μL of forward and reverse primers (10 μM; Table 1), 0.06 μL of PCV3-probe (100 μM), 2 μL of the template, and ddH_2_O to a final volume of 20 μL. The amplification program was 95°C for 7 min, 96°C for 10 s, and 60°C for 30 s, repeated for 45 cycles. The PCR reactions were performed on a Quant Studio 3 (Thermo Fisher Scientific, USA).

The primers and probes used in this study were based on established protocols [38, 39]. To account for interlaboratory variability in Ct thresholds, detection criteria were standardized using recombinant plasmids (pEASY-PCV2 and pCC1-4k-PCV3) constructed from full-length PCV2 (ON359945) and PCV3 (KT869077) genomes as positive controls, with ddH_2_O as the negative control. Standard curves were generated from tenfold serial dilutions (10^9^–10^0^ copies/μL). A positive threshold of Ct below 37 was established based on reproducibility validation and published references [38, 39] to ensure consistency and reliability of the detection results.

### 2.4. Conventional PCR Amplification and Sequencing

A total of four pairs of primers were used for the amplification of the PCV2 and PCV3 genomes (Table 1). The PCR conditions were as follows: 20 µL of 2 × Phanta Max Master Mix (Vazyme Biotech, Shanghai, China), 2 µL of 10 µM of each primer, 2 µL of the template, and ddH_2_O to bring the final volume up to 40 µL. The cycling conditions were 95°C for 5 min followed by 40 cycles of 95°C for 30 s, 55°C for 15 s, 72°C for 30 s, and a final extension at 72°C for 5 min. All PCR products were detected using 1% agarose gel electrophoresis. The products were then purified using Gel Extraction Kit (Omega Bio-tek, Norcross, USA) and sent to Tsingke Company (Nanjing, China) for Sanger sequencing. The obtained gene sequences were assembled using SeqMan v5.06 (DNASTAR, Madison, Wisconsin, USA).

### 2.5. Phylogenetic Analysis and Statistical Analysis

The sequences of PCV2 and PCV3 obtained in this experiment, along with other reference sequences (Supporting Information 1: Table S2), were aligned using ClustalW. The *ORF2* of PCV2 was then used to construct a phylogenetic tree employing the maximum likelihood method with 1000 replicates, using in MEGA X software (Mega Limited, New Zealand). A phylogenetic tree based on *PCV3 ORF2* sequence was constructed using the same method as above.

The PCV2 and PCV3 qPCR positive detection rates of different samples were compared using logistic regression, the effect of sample type on Ct values was evaluated using Kruskal–Wallis tests. *p*-values lower than 0.05 were considered statistically significant. Analyses were conducted using SPSS Statistics 26.0 software (IBM, Chicago, USA).

## 3. Results

### 3.1. qPCR Results of PCV2 and PCV3 Detection

A total of 132 samples were tested for PCV2 and PCV3 using TaqMan qPCR. These included 44 pork samples, 46 cutting board swabs, and 42 meat stall floor swabs, all collected from 46 meat stalls across six border cities in northeast China. The standard curves indicated that the qPCR amplification efficiencies were 109% (slope = 3.123) for PCV2 and 101% (slope = 3.288) for PCV3 with correlation coefficient (R^2^) both greater than 0.99. Detailed results, including standard curves, sample types, sources, and PCR Ct values, are provided in Supporting Information 2: Table S1 and Supporting Information 3: Figures S1 and S2. The positive cutoff thresholds (Ct <37) for PCV2 and PCV3 were validated, aligning previously published references [38, 39]. For PCV2, the highest detection rate of 75.0% (95% CI: 59.7%−86.8%) was found in pork samples, followed by 73.9% (95% CI: 58.9%−85.7%) in cutting board swabs and 64.3% (95% CI: 48.0%−78.4%) in meat stall floor swabs (Table 2). However, logistic regression analysis indicated that the detection rate in pork samples was not significantly higher than that in cutting board swabs(*p*=0.906) or meat stall floor swabs (*p*=0.281; Table 2).

For PCV3, the highest detection rate was found in cutting board swabs at 67.4% (95% CI: 52.0%−80.5%), followed by 54.7% (95% CI: 38.7%−70.2%) in meat stall floor swabs and 31.8% (95% CI: 18.6%−47.6%) in pork samples. Logistic regression analysis for PCV3 detection rates showed that the odds of detecting PCV3 in pork samples were significantly lower than in cutting board swabs samples (*p*=0.001; Table 2).

Kruskal–Wallis tests on the Ct values revealed that the sample type had no significant effects on the distribution of Ct values for PCV2-positive samples (*p*=0.066). Similarly, the distribution of Ct values for PCV3-positive sample was not significantly affected by sample type (*p*=0.41).

**Figure 1:**
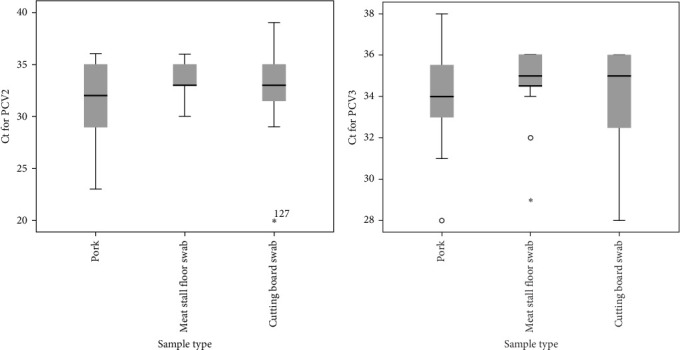
(a, b) Box plot showing the distribution of Ct values for PCV2 and PCV3-positive samples across different sample types (pork, cutting board swabs, and meat stall floor swabs). The horizontal line within each box represents the median, while the top and bottom of the box indicate the 75th and 25th percentiles, respectively. Whiskers represent the range, excluding outliers, which are shown as individual points. *⁣*^*∗*^ denotes extreme outliers.

For PCV2, pork samples exhibit a wide distribution of Ct values with a median in the midrange, while meat stall floor samples show less variability and a lower median. The box height for cutting board swabs is intermediate between pork and meat stall floor swabs, with the median in the middle range (Figure 1). For PCV3, meat stall floor swabs exhibit extreme outliers and a lower median, indicating a more dispersed distribution despite a relatively short interquartile range (Figure 1).

**Figure 2:**
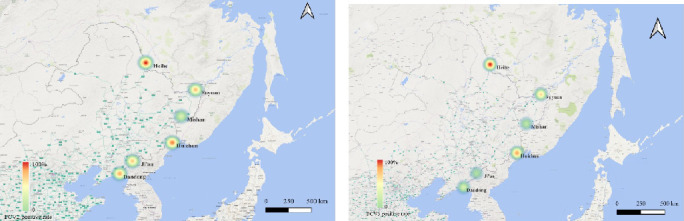
Heat map showing the positive rate of all samples types (pork, cutting board swabs, and meat stall floor swabs) for PCV2 (a) and PCV3 (b) from six sampling city. The color from green to red represents the positive rate from high to low.

Samples collected in Heihe City in Heilongjiang Province had the highest detection rates, with 85.2% testing positive for PCV2 and 70.4% for PCV3. The regional distribution of positive samples is illustrated in Figure 2.

### 3.2. Phylogenetic Analysis of *PCV2 ORF2*

Five *PCV2 ORF2* gene sequences were successfully obtained through conventional PCR amplification, all of which were derived from pork samples. Although some cutting board and meat stall floor swabs exhibited low Ct values in the TaqMan qPCR, the *PCV2 ORF2* gene could not be amplified from these samples using conventional PCR (Table 3).

**Figure 3:**
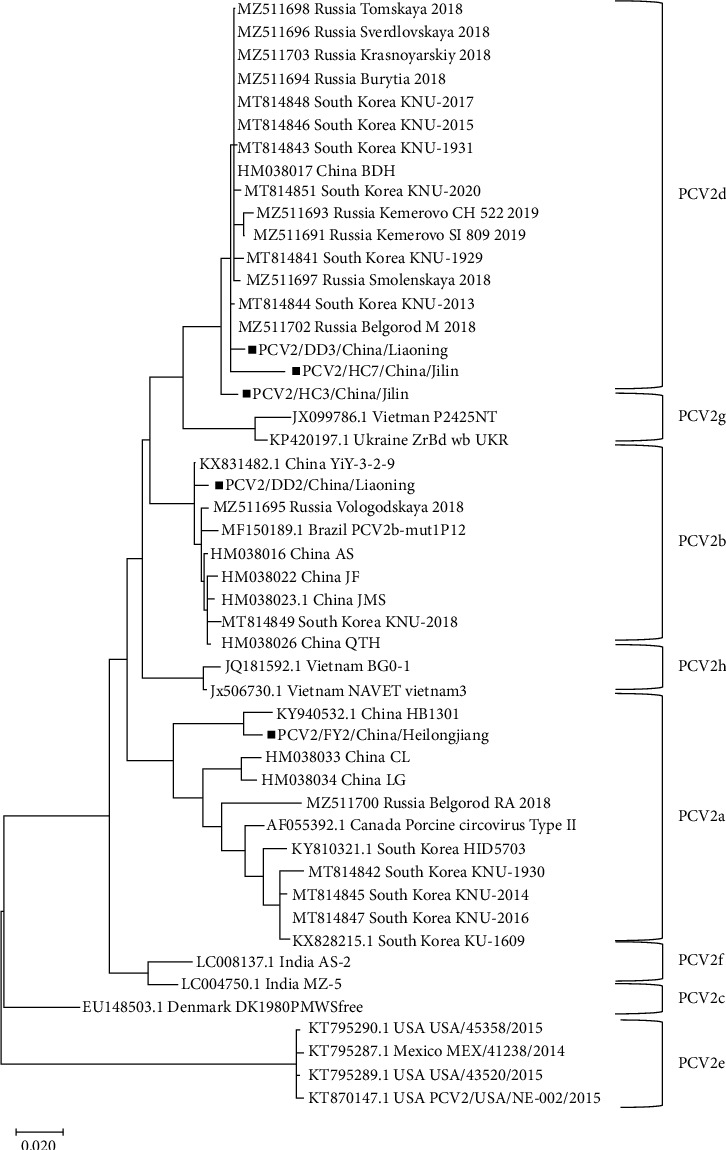
Phylogenetic trees based on *ORF2* genome of PCV2. Five *PCV2 ORF2* genomes generated in this study marked with a solid black square.

These *ORF2* gene sequences were subsequently analyzed using phylogenetic methods and compared with reference sequences obtained from GenBank (Supporting Information 1: Table S2). The analysis revealed that the sequences PCV2/HC3/China/Jilin, PCV2/HC7/China/Jilin, and PCV2/DD3/China/Liaoning belong to the PCV2d genotype. Meanwhile, PCV2/DD2/China/Liaoning was classified as PCV2b and PCV2/FY2/China/Heilongjiang was identified as PCV2a (Figure 3).

**Figure 4:**
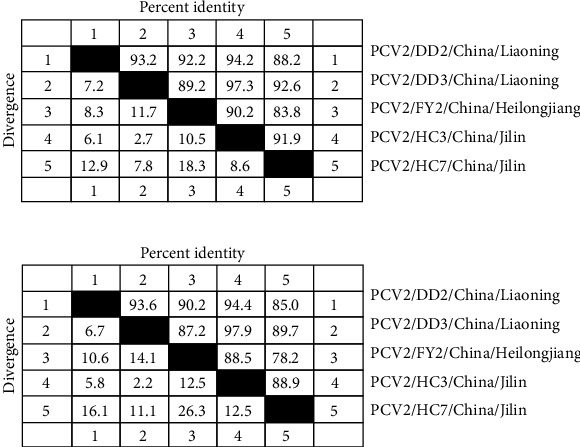
Nucleotide (a) and amino acids (b) homology analysis of *PCV2 ORF2* gene sequences collected in this study.

The nucleotide and amino acid sequences of the five obtained PCV2 Cap proteins were compared (Figure 4). The nucleotide similarity among the five sequences ranged from 83.8% to 97.3%, with the highest similarity of 97.3% observed between PCV2/DD3/China/Liaoning and PCV2/HC3/China/Jilin. The amino acid similarity among the five sequences ranged from 78.2% to 97.9%.

Further analysis showed that the PCV2/DD3/China/Liaoning gene sequence shares 99.4% homology with a strain found in Russia (MZ511702). The PCV2/DD2/China/Liaoning shares 99.4% homology with a PCV2 strain *ORF2* found in Henan, China (KX831482), and 98.9% homology with a strain found in Russia (MZ511695). PCV2/FY2/China/Heilongjiang shares 98.1% homology with a PCV2 strain *ORF2* found in Hebei, China (KY940532).

### 3.3. Phylogenetic Analysis of *PCV3 ORF2*

**Figure 5:**
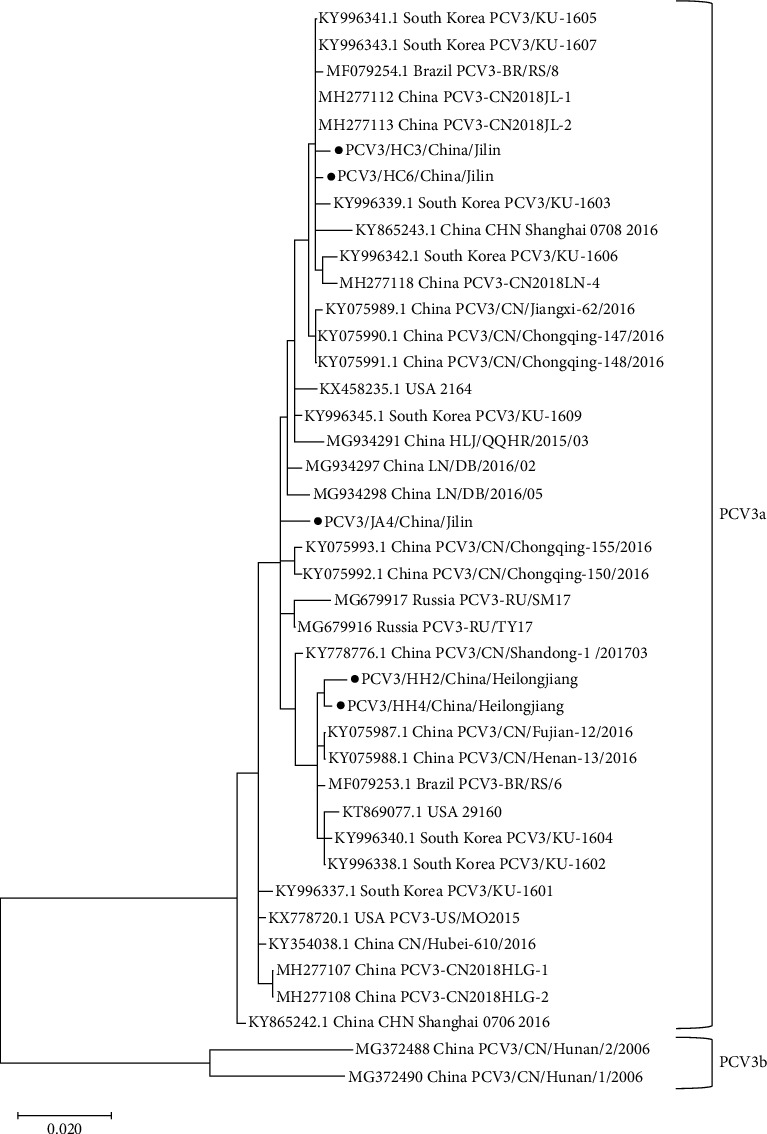
Phylogenetic trees based on *ORF2* genome of PCV3. Five *PCV3 ORF2* genomes generated in this study marked with a solid black circle.

Five *PCV3 ORF2* gene sequences were successfully obtained through conventional PCR amplification (Accession number: PQ684443–PQ684447), all from pork samples (Table 3). Based on previous study [24] and reference sequences obtained from GenBank (Supporting Information 1: Table S2), a phylogenetic tree based on *PCV3 ORF2* was constructed (Figure 5). By comparing the p-distance of *PCV3 ORF2*, two clades were identified: PCV3a and PCV3b, and five *ORF2* sequences obtained belong to the PCV3a genotype.

Based on the phylogenetic tree results, these two *PCV3 ORF2* sequences HC3/China/Jilin and HC6/China/Jilin had a homology of 99.6% and 99.8%, with sequences obtained from pig farms in Jilin, China (MH277112 and MH277113) and South Korea (KY996341.1 and KY996343.1).

**Figure 6:**
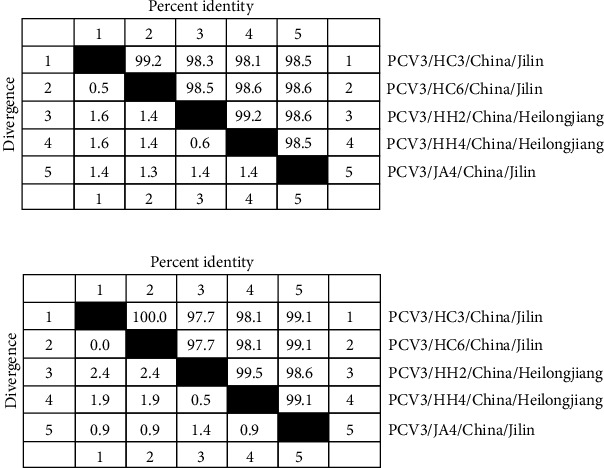
Nucleotide (a) and amino acids (b) homology analysis of *PCV3 ORF2* gene sequences collected in this study.

The nucleotide sequences of the five obtained PCV3 cap proteins were compared (Figure 6). The nucleotide similarity among the five sequences was above 98%, with the highest similarity of 99.2% observed between PCV3/HC3/China/Jilin and PCV3/HC6/China/Jilin.

## 4. Discussion

To the best of our knowledge, this is the first study to investigate the presence of PCV2 and PCV3 in live markets across multiple Chinese border cities. Live markets, which are prominent trading venues for fresh meat in China, obtain meat from a wide range of sources, including various producers and slaughterhouses. Evidence suggests that the trade of animals and animal products can facilitate the transmission of various infectious zoonotic and epizootic diseases, with the risk of spread increasing proportionally with trade volume [42]. During outbreaks of emerging or reemerging swine diseases, live markets may serve as critical sites for pathogen detection and regional outbreak assessment. Moreover, they may act as early-warning sentinels for transboundary animal diseases. Despite their importance, active surveillance of swine pathogens in live markets remains inadequate in China—particularly in the northeastern border regions, which are characterized by long international borders spanning three provinces. Continuous surveillance in these areas is often constrained by the considerable manpower and financial input required. Nonetheless, our results highlight the practicality and feasibility of detecting PCV2 and PCV3 from live market environments, supporting the use of such sites for swine pathogens monitoring and contributing to early warning systems for disease control.

The sampling sites in this study encompassed six border cities located in northeastern China, spanning three provinces and adjacent to two neighboring countries. Border cities are at the intersection of two countries, where trade is popular due to price differences in goods [43, 44]. Recently, because of visa-free policy, people from China and Russia frequently buy fresh food and goods in the live markets [45]. This is particularly evident in Heihe, which is located across the river from Blagoveshchensk, the third-largest city in Russia's far east, where close economic and cultural exchanges occur between the two cities. Notably, the samples collected in Heihe had a high positive rate for PCV2 and PCV3 detected by qPCR. The location of sampling sites in border-proximate live markets implies that cross-border trade and the high frequency of human movement may significantly contribute to the increased risk of viral transmission [46, 47].

The detection rate of PCV3 in cutting board swabs was higher than that in both pork samples and meat stall floor swabs, and the detection rate of PCV2 in cutting board swabs was second only to pork samples, while logistic regression analysis showed no significant differences. A higher detection rate of PCV2 and PCV3 may be attributed to the role of cutting boards as the primary surface for handling and processing meat, where pathogens from pork are readily transferred. The repeated contact of the cutting board with various pork products, along with the accumulation of blood and meat residue on its surface, and the porous structure of wood, likely create a conductive environment for the microbial survival and proliferation [48, 49]. Cutting boards are shared between different pieces of meat, knives, and hands, and the repeated contact between cutting boards, pork products, and these tools may facilitate the spread of PCV2 and PCV3. In contrast, environment surfaces are frequently exposed to cleaning agents, disinfectants, and varying environmental conditions, which may reduce viral concentrations. The lower detection rate of PCV2 and PCV3 in meat stall floor swabs compared to cutting board swabs in this study suggests that daily environment disinfection and cleaning practices required by live markets management policy in China [37] may contribute to controlling virus presence.

Although some cutting board swabs showed high positive detection rates for PCV2 and PCV3 via TaqMan qPCR, and several exhibited low Ct values, conventional PCR failed to amplify *ORF2* gene sequences from these samples. In contrast, all viral sequences obtained in this study were successfully amplified from pork samples. While qPCR is highly sensitive and specific for detecting viral nucleic acids, it cannot differentiate between infectious and noninfectious viral particles [50]. Moreover, previous studies have shown that viral inactivation, such as that of coronaviruses, occurs more rapidly on porous surfaces [51], and environmental disinfectants can degrade viral nucleic acids [52], potentially compromising the integrity of the viral genome in swab samples. These findings suggest that surface swabs may not preserve complete viral RNA or DNA adequately for subsequent amplification. Our study recommends that pork samples are the most appropriate sample type among those tested for the successful recovery of PCV2 and PCV3 genomes.

The *ORF2* sequences of the PCV2 and PCV3 obtained in this study classified into different subtypes, indicating a diverse origin of viral strains present in live markets within border cities. Notably, the high genetic homology of PCV2 and PCV3 across different regions suggest potential dissemination pathways along major pork trade routes. Specifically, the PCV2/DD2/China/Liaoning strain shared 99.4% nucleotide identity with the reference strain from Henan province (KX831482), while the PCV2/FY2/China/Heilongjiang isolate displayed 98.1% sequence similarity to the strain originating from Hebei province (KY940532); in addition to exhibiting a high nucleotide homology between the two sequences, their amino acid sequences also demonstrated substantial similarity. As shown in Figure S3, the two *PCV2 ORF2* sequences obtained in this study exhibited mutations at amino acid positions 130 and 133, which differed from those observed in other reference sequences. These mutations are located within the second B cell-defined immunogenic epitope of the capsid protein, a key factor influencing PCV2 virulence [53]. Amino acid changes in this epitope may lead to antigenic variation and immune evasion of PCV2 [54]. The two *PCV3 ORF2* sequences obtained in Heihe, Heilongjiang (PCV3/HH2/China/Heilongjiang and PCV3/HH4/China/Heilongjiang) had 99.2% and 99.5% homology with a *PCV3 ORF2* strain found in Fujian, China (KY075987). Amino acid alignment analysis revealed that the two *PCV3 ORF2* sequences obtained in this study share identical residues, with the reference strain KY075987 at positions 77 and 147 (Figure S4). Furthermore, the PCV3/HH2/China/Heilongjiang sequence exhibits amino acid mutations at positions 203 and 204, which were previously predicted to reside within B cell epitope regions [55]. While the functional implications of these mutations remain unclear, their potential impact on viral transmissibility and antigenicity warrants further investigation. Collectively, these findings underscore the close genetic relatedness of PCV2 and PCV3 strains circulating across different regions of China, likely driven by cross-regional movement within the pork supply chain. Further research, including tracing the source pig farms and specific transport routes of pork, is needed to determine the transmission network of PCV2 and PCV3 and to develop effective control strategies.

This study analyzed 132 samples collected from six border cities, including 44 pork samples, 46 cutting board swabs, and 42 meat stall floor swabs, to assess the presence of PCV2 and PCV3. A total of five *PCV2 ORF2* gene sequences and five *PCV3 ORF2* gene sequences were successfully obtained. Owing to challenges in manpower and logistics, the total number of samples collected from pork shops was relatively small in comparison to other surveillance studies conducted on farms or at slaughterhouses. Additionally, this study employed a cross-sectional design, assessing the virus-positive rate at a single time point in live markets. Unlike a cohort study, single time-point sampling may fail to capture seasonal fluctuations or long-term trends. Nevertheless, our study was designed to provide an initial assessment of PCV2 and PCV3 contamination in border markets, serving as a baseline for future surveillance. Follow-up studies incorporating longitudinal sampling will be essential to gain a deeper understanding of seasonal dynamics and long-term trends.

Given the epidemiological and genetic analysis in this study, live markets in border cities can serve as crucial sentinel points along the winding and complex borderlines. Regular monitoring of pathogen transmission in these markets in the future can provide early warnings of emerging diseases, effectively trace the sources of pathogens, and facilitate the development of targeted control measures.

## Figures and Tables

**Table 1:** Primers used for PCV2 and PCV3 detection in the conventional PCR, TaqMan qPCR, and complete genome sequencing in this study.

Primer and probe	Sequence (5–3′)	Assay	Reference
PCV2-qF	GARACTAAAGGTGGAACTGTACC	qPCR	[39]
PCV2-qR	TCCGATARAGAGCTTCTACAGC		
PCV2-probe	VIC-AGGAGTACCATTCCAACGGGG-BHQ1		
PCV3-qF	TGACGGAGACGTCGGGAAAT		[38]
PCV3-qR	CGGTTTACCCAACCCCATCA		
PCV3-probe	FAM-GGGCGGGGTTTGCGTGATTT-BHQ1		
PCV2-over-F	TTTCCGCGGGCTGGCTGAACTTTTGAAAGT	Conventional PCR complete genome sequencing	[40]
PCV2-over-R	AGCCCGCGGAAATTTCTGACAAACGTTACA		
PCV3-F1	CACCGTGTGAGTGGATATAC		[41]
PCV3-R1	CACCCCAACGCAATAATTGTA		
PCV3-F2	TTGGGGTGGGGGTATTTATT		
PCV3-R2	ACACAGCCGTTACTTCAC		
PCV3-F3	AGTGCTCCCCATTGAACG		
PCV3-R3	CGACCAAATCCGGGTAAGC		

**Table 2:** Detection of Porcine circovirus type 2 and 3 by real-time quantitative polymerase chain reaction (qPCR) across different sample types.

Sample type	Pork	Meat stall floor swab	Cutting board swab
PCV2 detection rate (95% CI)	75.0% (59.7%−86.8%)	64.3% (48.0%−78.4%)	73.9% (58.9%−85.7%)
PCV2 odds ratio (95% CI)	1 (reference group)	0.600 (0.24–1.52)	0.94 (0.37–2.44)
PCV3 detection rate (95% CI)	31.8% (18.6%−47.6%)	54.7% (38.7%−70.2%)	67.4% (52.0%−80.5%)
PCV3 odds ratio (95% CI)	0.226 (0.09–0.55)	0.586 (0.25–1.39)	1 (reference group)

**Table 3:** Sequence information on *PCV2* and *PCV3 ORF2* gene sequences collected in this study.

Sequence name	City of sample collection	PCV2 Ct values	PCV3 Ct values
PCV2/DD2/China/Liaoning	Dandong	28	—
PCV2/DD3/China/Liaoning	Dandong	29	—
PCV2/HC3/China/Jilin	Hunchun	23	—
PCV2/HC7/China/Jilin	Hunchun	30	—
PCV2/FY2/China/Heilongjiang	Fuyuan	26	—
PCV3/JA4/China/Jilin	Ji'an	—	34
PCV3/HC3/China/Jilin	Hunchun	—	28
PCV3/HC6/China/Jilin	Hunchun	—	32
PCV3/HH2/China/Heilongjiang	Heihe	—	31
PCV3/HH4/China/Heilongjiang	Heihe	—	32

## Data Availability

All the data supporting the findings of this study are included in the article and its supporting information.

## Supporting Information

Supporting Information 1 Table S2 GenBank accession number of the reference sequences.

Supporting Information 2 Table S1 Information on sample types, sources, and data results.

Supporting Information 3 Figure S1 Standard curve of PCV2 qPCR by serial dilutions of cloned control constructs. Figure S2 Standard curve of PCV3 qPCR by serial dilutions of cloned control constructs. Figure S3 Amino acid alignment of ORF2 proteins from PCV2 strains. Figure S4 Amino acid alignment of ORF2 proteins from PCV3 strains.
