# Supplementary material for: Investigating the Presence and Genetic Variability of Porcine Circovirus Types 2 and 3 in Live Markets in Border Cities of Northeast China
Source: Transbound Emerg Dis. 2025 Jun 19;2025:5526645. doi: 10.1155/tbed/5526645 (PMC12202078; doi:10.1155/tbed/5526645)
Supplement: Supporting Information 1 — Table S2 GenBank accession number of the reference sequences. [file 5526645.f1.docx]

**Supplementary Table S2:** Genbank accession number of the reference sequences

| Genotype | Accession No. | Genotype | Accession No. |
| --- | --- | --- | --- |
| PCV2d | MZ511698 | PCV3a | KT869077.1 |
| PCV2d | MZ511696 | PCV3a | NC031753.1 |
| PCV2d | MZ511703 | PCV3a | KY996340.1 |
| PCV2d | MZ511694 | PCV3a | KY996338.1 |
| PCV2d | MT814848 | PCV3a | MF079253.1 |
| PCV2d | MT814846 | PCV3a | KY075987.1 |
| PCV2d | MT814843 | PCV3a | KY075988.1 |
| PCV2d | HM038017 | PCV3a | KY778776.1 |
| PCV2d | MT814851 | PCV3a | KY075993.1 |
| PCV2d | MZ511693 | PCV3a | KY075992.1 |
| PCV2d | MZ511691 | PCV3a | MG679917 |
| PCV2d | MT814841 | PCV3a | MG679916 |
| PCV2d | MZ511697 | PCV3a | KY865242.1 |
| PCV2d | MT814844 | PCV3a | KY996337.1 |
| PCV2d | MZ511702 | PCV3a | KX778720.1 |
| PCV2g | JX099786.1 | PCV3a | KY354038.1 |
| PCV2g | KP420197.1 | PCV3a | MH277107 |
| PCV2b | KX831482.1 | PCV3a | MH277108 |
| PCV2b | MZ511695 | PCV3a | MG934297 |
| PCV2b | MF150189.1 | PCV3a | MG934298 |
| PCV2b | HM038016 | PCV3a | KY996345.1 |
| PCV2b | MT814849 | PCV3a | KX458235.1 |
| PCV2b | MT81485 | PCV3a | KY075991.1 |
| PCV2b | HM038022 | PCV3a | KY075989.1 |
| PCV2b | HM038023.1 | PCV3a | KY996342.1 |
| PCV2b | HM038026 | PCV3a | MH277118 |
| PCV2h | JQ181592.1 | PCV3a | KY865243.1 |
| PCV2h | JX506730.1 | PCV3a | MH277112 |
| PCV2a | KY940532.1 | PCV3a | MH277113 |
| PCV2a | HM038033 | PCV3a | MF079254.1 |
| PCV2a | HM038034 | PCV3a | KY996341.1 |
| PCV2a | MZ511700 | PCV3a | KY996343.1 |
| PCV2a | AF055392.1 | PCV3a | MG934291 |
| PCV2a | KY810321.1 | PCV3b | MG372488.1 |
| PCV2a | MT814842 | PCV3b | MG372490.1 |
| PCV2a | MT814845 |  |  |
| PCV2a | MT814847 |  |  |
| PCV2a | KX828215.1 |  |  |
| PCV2f | LC008137.1 |  |  |
| PCV2f | LC004750.1 |  |  |
| PCV2c | EU148503.1 |  |  |
| PCV2e | KT795290.1 |  |  |
| PCV2e | KT795287.1 |  |  |
| PCV2e | KT795289.1 |  |  |
| PCV2e | KT870147.1 |  |  |
