# Supplementary material for: Investigating the Presence and Genetic Variability of Porcine Circovirus Types 2 and 3 in Live Markets in Border Cities of Northeast China
Source: Transbound Emerg Dis. 2025 Jun 19;2025:5526645. doi: 10.1155/tbed/5526645 (PMC12202078; doi:10.1155/tbed/5526645)

**Supplementary Figure S1:** Standard curve of PCV2 qPCR by serial dilutions of cloned control constructs.


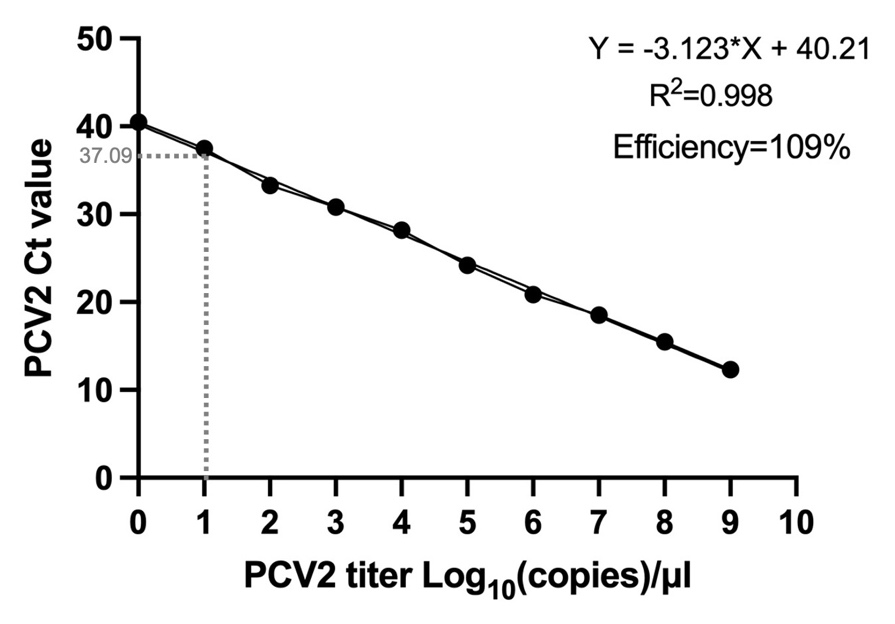


**Supplementary Figure S2:** Standard curve of PCV3 qPCR by serial dilutions of cloned control constructs.

**
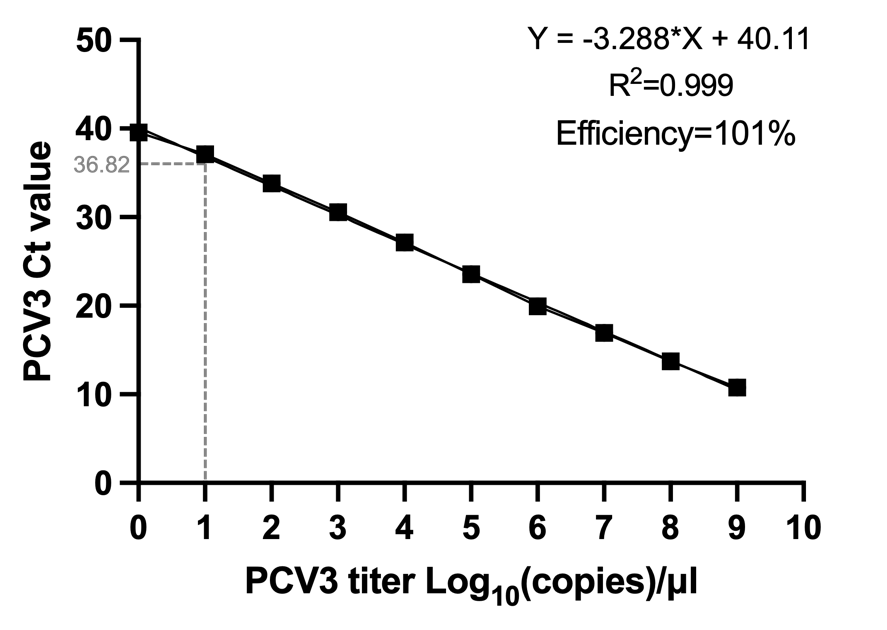
**

**Supplementary Figure S1:** Amino acid alignment of ORF2 proteins from PCV2 strains.


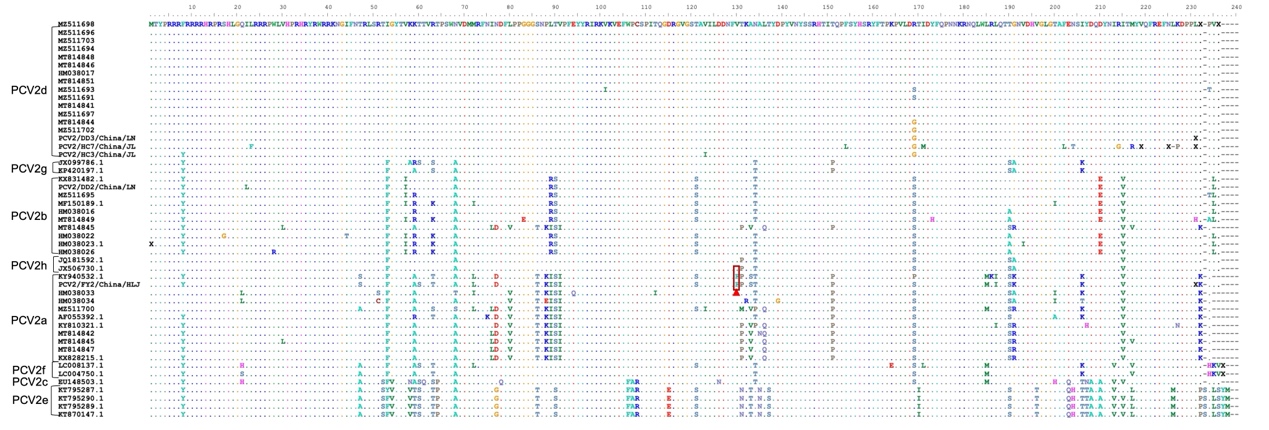


**Supplementary Figure S2:** Amino acid alignment of ORF2 proteins from PCV3 strains.


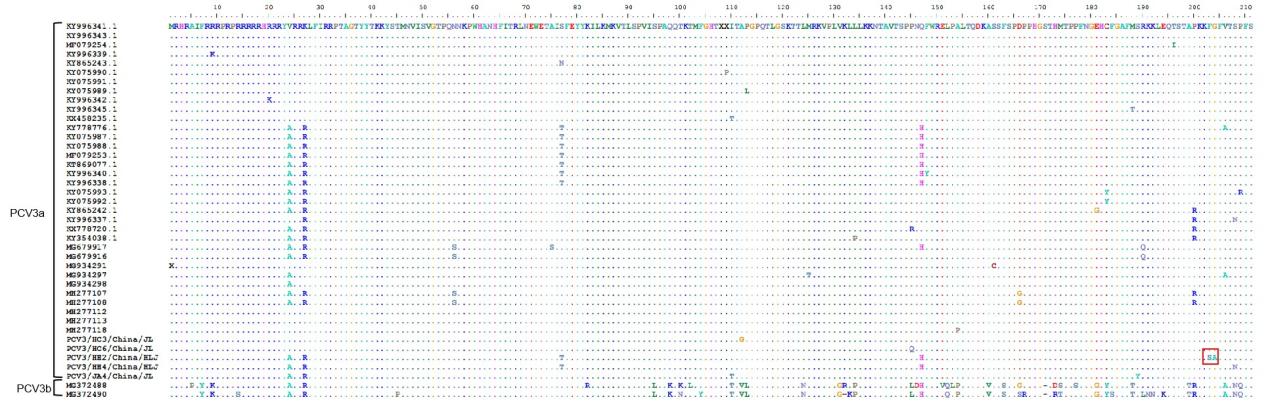

Supplement: Supporting Information 3 — Figure S1 Standard curve of PCV2 qPCR by serial dilutions of cloned control constructs. Figure S2 Standard curve of PCV3 qPCR by serial dilutions of cloned control constructs. Figure S3 Amino acid alignment of ORF2 proteins from PCV2 strains. Figure S4 Amino acid alignment of ORF2 proteins from PCV3 strains. [file 5526645.f3.docx]
